# The emerging role of NOTCH3 receptor signalling in human lung diseases

**DOI:** 10.1017/erm.2022.27

**Published:** 2022-09-02

**Authors:** Manish Bodas, Bharathiraja Subramaniyan, Harry Karmouty-Quintana, Peter F. Vitiello, Matthew S. Walters

**Affiliations:** 1 Department of Medicine, Section of Pulmonary, Critical Care & Sleep Medicine, University of Oklahoma Health Sciences Center, Oklahoma City, OK 73104, USA; 2 Department of Biochemistry and Molecular Biology, McGovern Medical School, University of Texas Health Science Center at Houston, Houston, Texas 77030, USA; 3 Department of Internal Medicine, Divisions of Critical Care, Pulmonary and Sleep Medicine, McGovern Medical School, University of Texas Health Science Center at Houston, Houston, Texas 77030, USA; 4 Department of Pediatrics, University of Oklahoma Health Sciences Center, Oklahoma City, OK 73104, USA

**Keywords:** Asthma, COPD, human lung disease, IPF, lung cancer, NOTCH3 signalling, PAH, viral infections

## Abstract

The mammalian respiratory system or lung is a tree-like branching structure, and the main site of gas exchange with the external environment. Structurally, the lung is broadly classified into the proximal (or conducting) airways and the distal alveolar region, where the gas exchange occurs. In parallel with the respiratory tree, the pulmonary vasculature starts with large pulmonary arteries that subdivide rapidly ending in capillaries adjacent to alveolar structures to enable gas exchange. The NOTCH signalling pathway plays an important role in lung development, differentiation and regeneration post-injury. Signalling via the NOTCH pathway is mediated through activation of four NOTCH receptors (NOTCH1-4), with each receptor capable of regulating unique biological processes. Dysregulation of the NOTCH pathway has been associated with development and pathophysiology of multiple adult acute and chronic lung diseases. This includes accumulating evidence that alteration of NOTCH3 signalling plays an important role in the development and pathogenesis of chronic obstructive pulmonary disease, lung cancer, asthma, idiopathic pulmonary fibrosis and pulmonary arterial hypertension. Herein, we provide a comprehensive summary of the role of NOTCH3 signalling in regulating repair/regeneration of the adult lung, its association with development of lung disease and potential therapeutic strategies to target its signalling activity.

## Introduction

**Fig. 1.**
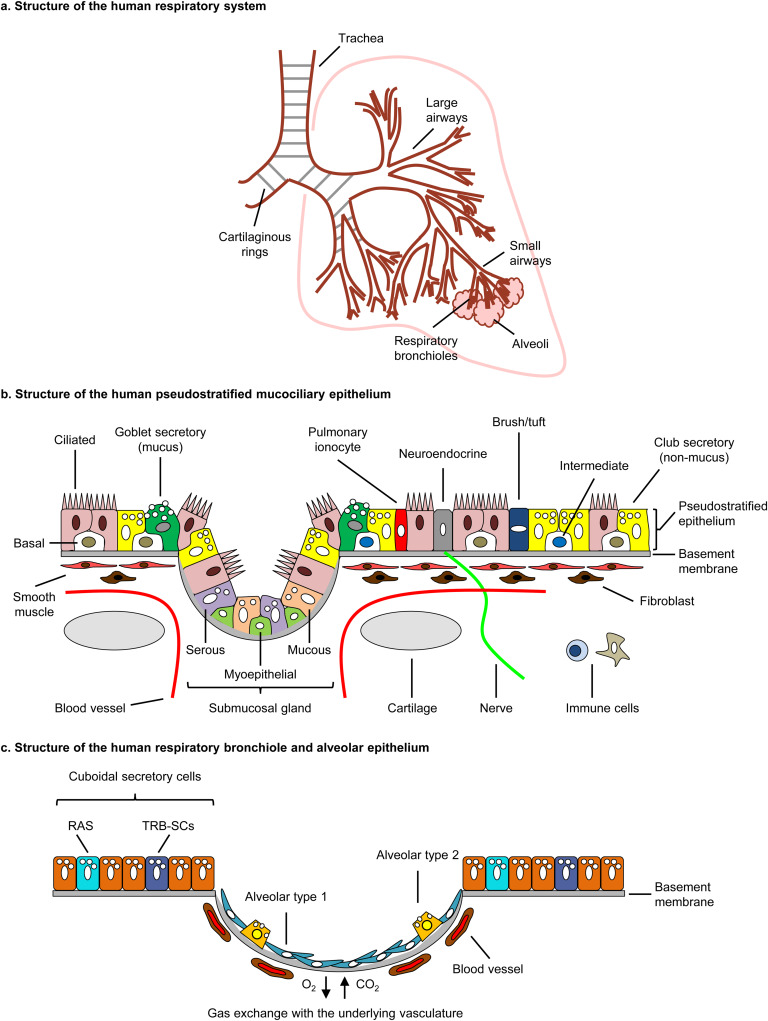
The human respiratory system. (a) Structure of the human respiratory system. (b) Structure of the human pseudostratified mucociliary epithelium. The pseudostratified mucociliary airway epithelium is a continuous single layer of epithelial cells with each cell having direct contact with the basement membrane. The mucociliary epithelium consists of several cell types which can be identified by expression of specific markers. These include, basal cells (KRT5 + , TP63 + ), intermediate (KRT8 + , KRT13 + ), multi-ciliated (FOXJ1 + , DNAI1 + ), goblet secretory (MUC5AC + , MUC5B + ), club secretory (SCGB1A1 + , SCGB3A2 + ), neuroendocrine (CALCA + , ASCL1 + ), pulmonary ionocytes (FOXI1 + , CFTR + ) and brush/tuft (TSLP + , IL-25 + ). The trachea and large airways also harbour submucosal glands which contain additional specialised epithelial cell populations including, serous (LTF + , DCCP1 + ), mucous (MUC5B + , TFF2 + ) and myoepithelial (EPCAM + , ACTA2 + ). Located underneath the basement membrane are a large number of non-epithelial cell populations required to maintain proper structure and function of the respiratory system, including cartilage rings (trachea and large airways only), smooth muscle, fibroblasts, blood vessels, nerves and immune cells (e.g., lymphocytes and dendritic cells). (c) Structure of the human respiratory bronchiole and alveolar epithelium. Terminal respiratory bronchioles are lined predominantly with cuboidal secretory cells (SCGB1A1 + , SCGB3A2 + ) which contain the recently identified progenitor populations termed respiratory airway secretory (RAS) or terminal and respiratory bronchiole secretory cells (TRB-SCs). In contrast, the alveoli consist of alveolar type 1 (AGER + , AQP5 + ) and type 2 (SFTPC + , ABCA3 + ) cells. Type 1 cells are the predominant epithelial cell type in the alveolus and comprise approximately 95% of the gas exchange surface with the underlying vasculature in the lung.

The mammalian respiratory system or lung is one of the most critical organ systems, and the main site of gas exchange with the external environment (Refs 1, 2, 3, 4, 5, 6, 7, 8). Structurally, the human lung is a tree-like branching structure which is broadly classified into the conducting airways (proximal and distal), which terminate at the respiratory bronchioles and form a connection with the alveolar region, where the gas exchange occurs (Fig. 1a) (Refs 1, 2, 3, 4, 5, 6, 7, 8). Similar to the respiratory tree, the pulmonary circulation subdivides rapidly and branches into capillaries that surround the alveolar compartment, allowing for a large surface area for gas exchange (Ref. 9). However, contrary to the airway tree, segments of the pulmonary artery branch off early at irregular but frequent intervals to enter the lung parenchyma. This results in the pulmonary arterial tree having more branches than the bronchial one (Refs 10, 11). Following gas exchange in the capillary beds, oxygenated blood is returned to the heart by pulmonary veins (Ref. 9).

The conducting airway, comprising of the nasal cavity, trachea, bronchi and bronchioles, functions as a conduit of air to and from the alveoli and is the foremost physical barrier and first line of defence against inhaled pathogens (e.g., viruses and bacteria) and particulates (Refs 1, 2, 3, 4, 5, 6, 7, 8). This efficient barrier is formed by the pseudostratified mucociliary airway epithelium, a continuous single layer of epithelial cells, with each cell having a direct contact to the basement membrane (Fig. 1b) (Refs 1, 2, 3, 4, 5, 6, 7, 8). Located underneath the basement membrane are a large number of non-epithelial cell populations required to maintain proper structure and function of the respiratory system, including cartilage rings (trachea and large airways only), smooth muscle, fibroblasts, blood vessels, nerves and immune cells (e.g., lymphocytes and dendritic cells) (Fig. 1b) (Refs 1, 2, 3, 4, 5, 6, 7, 8). The mucociliary epithelium consists of several cell types, such as basal cells (BCs), intermediate, multi-ciliated, secretory (mucus producing ‘goblet’; or non-mucus producing ‘club’ cells), neuroendocrine and many other rare cell types, such as pulmonary ionocytes and brush/tuft (Fig. 1b) (Refs 1, 2, 3, 4, 5, 6, 7, 8, 12). Both multi-ciliated and secretory cells are critical to the barrier function of the mucociliary epithelium, as they form the mucociliary escalator system, which helps cleanse the airways (Refs 1, 2, 3, 4, 5, 6, 7, 8). In this process, the inhaled particulates and pathogens trapped on the apical surface of the epithelium by secretory cell-derived mucins and defence-related molecules are removed from the airways in a retrograde manner by the action of multi-ciliated cells. In addition to the luminal secretory cell populations, the trachea and large airways also harbour submucosal glands which contain additional specialised epithelial cell populations including, serous, mucous and myoepithelial that contribute to the production of luminal mucus (Fig. 1b) (Refs 1, 2, 3, 4, 5, 6, 7, 8). BCs are the resident stem/progenitor cells of the adult mucociliary airway epithelium that are responsible for the normal turnover of airway epithelial cells during homeostasis, and the repair and regeneration of the airway epithelium following injury (Refs 1, 2, 3, 4, 5, 6, 7, 8, 13, 14, 15). In contrast to the conducting airways, the terminal respiratory bronchioles are predominantly lined with cuboidal secretory cells which contain the recently identified progenitor populations termed respiratory airway secretory or terminal and respiratory bronchiole secretory cells (Fig. 1c) (Refs 16, 17). Upon transition to the alveoli, barrier function is mediated by the alveolar type 1 (AT1) and type 2 (AT2) cells (Fig. 1c) (Refs 1, 2, 3, 4, 5, 6, 7, 8, 18). AT1 cells are the predominant epithelial cell type in the alveolus and comprise approximately 95% of the gas exchange surface of the lung, with their flattened, squamous morphology providing an ideal interface for gas exchange with the underlying vasculature (Refs 1, 2, 3, 4, 5, 6, 7, 8, 18). The cuboidal AT2 cells are responsible for production of surfactant which helps reduce surface tension in the alveolar region during respiration (Ref. 19). In addition, AT2 cells function as progenitor cells for AT1 cells, thus maintaining normal turnover of the alveolar epithelial cells during homeostasis and replenish cells which are lost after injury (Ref. 18). Despite the ability of both the mucociliary and alveolar epithelium to repair and regenerate via the action of a resident adult stem/progenitor cells (i.e., BCs and AT2, respectively), the underlying ‘niche microenvironment’ (i.e., non-epithelial cell populations and extracellular matrix) of the lung plays an important role in regulating the cell differentiation and regeneration response of the epithelium via the production of key paracrine signals (Refs 1, 2, 3, 4, 5, 6, 7, 8, 18). Most of our current understanding of the mechanisms by which the lung responds to and regenerates post-injury comes from using murine lung-injury models. Despite the high conservation of lung structure and cellular composition between the human and mouse respiratory system, there are important differences (Refs 1, 2, 3, 4, 5, 6, 7). These include restriction of BC in the cartilaginous rings and submucosal glands up to the trachea of the mouse lung as compared to humans, where these features extend to the distal airways. In addition, there is a reduced number of branches and complete lack of respiratory bronchioles in the mouse lung, which are the site of injury in many human lung diseases and contain distinct secretory cell populations that function as progenitors for AT2 cells (Refs 16, 17). Therefore, under certain conditions these differences may limit the translation of lung repair and regeneration mechanisms identified in mouse to the human lung. However, the abundance of genetic mouse models and the development of new technologies to assess cellular composition and the molecular responses (i.e., transcriptional and epigenetic) of specific cell types post-injury have yielded critical information and advanced our understanding of the basic mechanisms regulating lung homeostasis and regeneration (Refs 1, 2, 3, 4, 5, 6, 7).

Adult human lung disease inflicts a large socio-economic burden and is a leading cause of morbidity and mortality worldwide (Refs 20, 21, 22, 23). This includes acute lung disease in response to viral (e.g., influenza, rhinovirus (RV), SARS-CoV-2) or bacterial (e.g., Streptococcus, Haemophilus, Pseudomonas) infections and chronic respiratory diseases, such as chronic obstructive pulmonary disease (COPD), asthma, idiopathic pulmonary fibrosis (IPF), pulmonary arterial hypertension (PAH) (Refs 24, 25, 26, 27, 28, 29, 30, 31, 32, 33, 34, 35, 36, 37, 38, 39, 40, 41, 42). Many of these diseases have mortality rates comparable to most types of lung cancer (Ref. 43). Development of both acute and chronic lung disease is associated with injury and alteration of the underlying architecture of the lung which disrupts its normal function (Refs 2, 3, 44). In the context of chronic lung disease, aberrant regeneration mechanisms lead to a failure to restore the normal architecture and cellular composition of the lung, which can eventually result in long-term lung function decline (Refs 2, 3, 44). Therefore, understanding the lung's response to injury and the mechanisms that regulate tissue repair and regeneration may help identify new therapeutic strategies to treat both acute and chronic lung disease.

The NOTCH signalling pathway plays an important role in lung development and regeneration of the adult lung post-injury (Refs 45, 46, 47, 48). Signalling via the NOTCH pathway is mediated through activation of four NOTCH receptors (NOTCH1–4), with each receptor capable of regulating unique biological processes (Ref. 49). Dysregulation of the NOTCH pathway has been associated with development and pathophysiology of multiple adult lung diseases including COPD, lung cancer, asthma, IPF and PAH (Refs 45, 48, 50, 51, 52, 53, 54, 55, 56, 57, 58, 59, 60, 61, 62, 63, 64, 65, 67, 68). However, the specific focus of this review will be to provide a comprehensive summary of the role of NOTCH3 receptor signalling in regulating repair/regeneration of the adult lung, its association with development of lung disease and potential therapeutic strategies to target its signalling activity.

## NOTCH signalling pathway

**Fig. 2.**
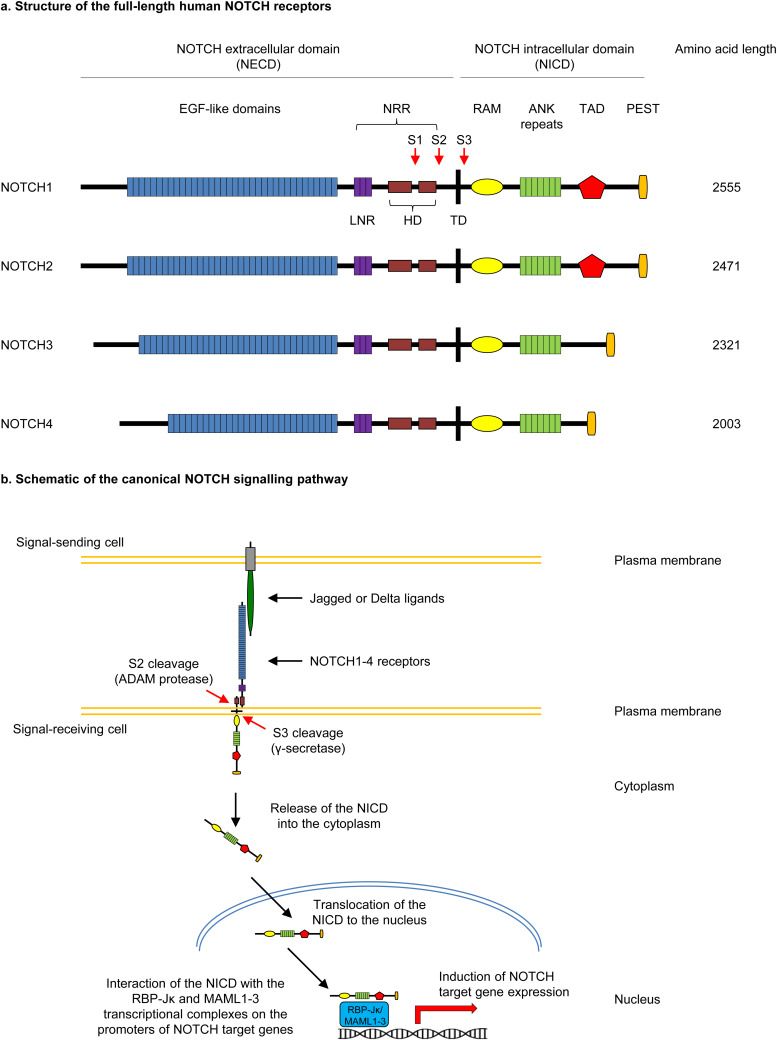
The NOTCH signalling pathway. (a) Structure of the full-length human NOTCH receptors. The four NOTCH receptors share a common structure consisting of a NOTCH extracellular domain (NECD), transmembrane domain (TD) and a NOTCH intracellular domain (NICD). The NECD contains approximately 29–36 epidermal growth factor (EGF)-like domains, a negative regulatory region (NRR) consisting of three Lin Notch repeats (LNR) and receptor heterodimerisation domains (HD). Following the TD, the NICD contains a RBP-J*κ*-association module (RAM) domain, ankyrin (ANK) repeats, a transactivation domain (TAD) and a C-terminal domain rich in proline, glutamic acid, serine and threonine (PEST) domain. The locations of the S1, S2 and S3 cleavage sites are indicated. (b) Schematic of the canonical NOTCH signalling pathway. Canonical NOTCH signalling transduction is relayed via cell-to-cell contact by the direct binding of cell-bound ligand to the NECD of a receptor on a neighbouring cell. Ligand binding results in activation of the NOTCH receptor on the signal-receiving cell via enzymatic cleavage at site 2 (S2) and site 3 (S3) on the NOTCH receptor via the ADAM protease and *γ*-secretase enzyme, respectively. Following cleavage of the NICD from the receptor and its release into the cytoplasm, the NICD translocates to the nucleus and interacts with transcriptional complexes (RBP-J*κ* and MAML1–3) on the promoters of NOTCH target genes, resulting in their transcription.

NOTCH signalling is a highly conserved cell-cell interaction signalling pathway that plays crucial roles in the development, repair and regeneration processes in the embryonic and adult lung (Refs 45, 46, 47, 48), as well as other organ systems (Ref. 49). The mammalian NOTCH signalling pathway consists of four transmembrane receptors (NOTCH1–4) and five ligands of the Jagged (Jagged1,2 – orthologues to fly Serrate) and Delta-like (Dll1,3,4 – orthologues to fly Delta) families (Refs 49, 69). The four NOTCH receptors share a common structure consisting of a transmembrane domain and a NOTCH extracellular domain (NECD) that is non-covalently associated with a NOTCH intracellular domain (NICD) which creates a hetero-dimeric, single-pass, transmembrane receptor (Fig. 2a) (Refs 49, 69). The NECD contains approximately 29–36 epidermal growth factor-like domains (which enables ligand interactions) and a negative regulatory region (NRR) consisting of three Lin Notch repeats and receptor heterodimerisation domains (HD) (Refs 49, 70). Similarly, the NICD contains multiple domains which regulate its transcriptional activity including an RBP-J*κ*-association module domain, ankyrin repeats, a transactivation domain (TAD) and a C-terminal domain rich in proline, glutamic acid, serine and threonine domain (Refs 49, 70). Full length NOTCH receptors are produced in the endoplasmic reticulum, and before trafficking to the plasma membrane they are proteolytically cleaved by the furin-like convertase (Site 1 or S1 cleavage) in the Golgi compartment to form a processed heterodimer (Fig. 2a) (Refs 49, 70). Canonical NOTCH signalling transduction is relayed via cell-to-cell contact by the direct binding of cell-bound ligand to the NECD of a receptor on a neighbouring cell (Fig. 2b) (Refs 49, 70). Ligand binding results in activation of the NOTCH receptor on the signal-receiving cell via multiple enzymatic cleavage events at site 2 (S2) and site 3 (S3) on the NOTCH receptor (Fig. 2a and b) (Refs 49, 70). S2 cleavage and release of the NECD occur via the ADAM protease, whereas S3 cleavage by the *γ*-secretase enzyme releases the NICD from the receptor (Ref. 49). This NICD translocates to the nucleus and interacts with other inactive transcriptional complexes (RBP-J*κ* and MAML1-3) resulting in the transcription of multiple NOTCH downstream target genes. These include the *HEY* and *HES* gene families which encode basic helix-loop-helix transcription factors that typically act as repressors of transcription (Fig. 2b) (Refs 49, 71). The large combination of ligand–receptor interactions and cell type-specific expression of pathway components makes the canonical NOTCH signalling pathway an exceptionally versatile system that can lead to highly dynamic and diverse downstream signalling responses (Ref. 49). In addition to canonical signalling, non-canonical NOTCH signalling has been reported whereby signalling occurs either independent of ligand interaction or RBP-J*κ* activation (Ref. 72). However, the role of non-canonical NOTCH signalling in lung development, repair and regeneration is poorly understood.

The human *NOTCH3* gene is located on chromosome 19p13.12 and encompasses 33 exons which encode for a protein composed of 2321 amino acids (Refs 56, 73, 74 75). Expression of *NOTCH3* has been found in multiple tissue types including the vasculature, smooth muscle, central nervous and immune system (Refs 56, 73, 74, 75). Relevant to the adult human and mouse lung, *NOTCH3* is expressed in vascular smooth muscle cells (VSMCs), pulmonary artery smooth muscle cells (PASMCs), pericytes, fibroblasts and specific cell types of the airway epithelium (e.g., basal-intermediate and club) (Refs 48, 51). While deletion of *Notch1* and *Notch2* is embryonic lethal in mice, deletion of *Notch3* does not affect embryo viability (Refs 45, 56). However, mice with deletion of *Notch3* have impairment of VSMC differentiation and maturation (including the lung) that leads to alterations in the vascular structure (Refs 45, 56). In addition, the tracheal pseudostratified airway epithelium of *Notch3^−/−^* mice have increased numbers of KRT8+ undifferentiated progenitor cells (i.e., intermediate cells) compared to wild-type mice suggesting that NOTCH3 signalling in the murine airway epithelium was critical for priming of BC differentiation into club cells (Ref. 51). In support of this, recent work from our lab demonstrated that NOTCH3 receptor signalling regulates BC to club cell differentiation in the human airway epithelium in vitro (Ref. 58). Lentivirus-mediated overexpression of the active NICD3 in primary human bronchial epithelial cells (HBECs) on in vitro air-liquid interface (ALI) culture promoted club cell differentiation. Furthermore, we demonstrated the NOTCH3 downstream target *HEYL* was important for regulating this process since siRNA-mediated knockdown of HEYL reduced club (SCGB1A1+), goblet (MUC5AC+) and ciliated cell (FOXJ1) differentiation, but lead to an increase in the number of KRT8+ intermediate cells. While mice with deletion of *Notch3* display no defects in the alveolar epithelium, over-expression of NICD3 during development inhibits terminal differentiation of the alveolar epithelium (Ref. 76). Despite sharing a similar structure to the NOTCH1 and NOTCH2 receptors, NOTCH3 displays a number of structural differences that may explain the unique aspects of NOTCH3 signalling and its regulation of lung biology compared to other NOTCH receptors (Fig. 2a) (Ref. 77). These include differences in the NOTCH3 ECD which potentially make the receptor more vulnerable to cleavage and activation in the absence of ligand (Ref. 77). In addition, the lack of a TAD in the NOTCH3 ICD may account for a weaker transactivation activity compared to other NOTCH ICDs.

Despite the knowledge NOTCH3 signalling is not essential for murine lung development, there is emerging evidence that dysregulation of NOTCH3 signalling in the adult human lung plays an important role in the development and pathogenesis of acute and chronic lung disease. Therefore, the remainder of this review will summarise recent new findings from ours and other groups, which highlight the pathogenic role of NOTCH3 in mediating severe respiratory diseases such as COPD, viral infections, lung cancer, asthma, IPF and PAH. Finally, we debate the potential and means of targeting NOTCH3 signalling as a therapeutic strategy for treatment of lung disease.

## NOTCH3 signalling and acute or chronic lung disease

### COPD and emphysema

COPD is a preventable, but life-threatening lung disease, and is the third leading cause of death in the United States (Ref. 20). Exposure to first and/or second-hand cigarette smoke (CS) is the leading risk factor for the initiation and progression of COPD pathophysiology, which is broadly classified into chronic bronchitis and emphysema (Refs 36, 78, 79). Chronic bronchitis is characterised by increased inflammation of the airways and excess mucus production which leads to airflow obstruction (Refs 36, 78, 79), whereas emphysema is a disease of the alveoli characterised by damage and permanent enlargement of the alveolar airspace, which reduces the surface area available for gas exchange (Refs 36, 78, 79).

CS exposure is known to mediate significant changes in the cellular architecture of the mucociliary airway epithelium (termed epithelial remodelling), including BC hyperplasia, squamous metaplasia, loss of club cells and increased numbers of mucus-producing goblet cells termed ‘goblet cell metaplasia or hyperplasia’ (GCMH) (Refs 14, 78, 79). Prior studies have identified alterations at the mRNA, protein and epigenetic level for multiple NOTCH signalling pathway components in the in vivo airway epithelium of smokers with and without COPD relative to that of non-smokers (Refs 50, 51, 52, 53). This includes decreased NOTCH3 mRNA in the airway epithelium of smokers versus non-smokers (Ref. 52), and decreased NOTCH3 protein levels in the airway epithelium of COPD versus non-COPD controls (Ref. 51). While protein levels of activated NOTCH3 receptor (i.e., NICD3) were not assessed in these studies, these data suggest that reduced levels of NOTCH3 receptor (and its downstream signalling) may contribute to the development of airway epithelial remodelling associated with CS exposure and COPD (Refs 14, 78, 79). In support of this, our recent study observed decreased expression of the NOTCH3 downstream target *HEYL* in HBECs from COPD versus normal (non-COPD) donors which correlated with the impaired differentiation capacity of COPD HBECs on in vitro ALI culture (Ref. 58). Furthermore, we demonstrated that lentivirus-mediated overexpression of HEYL in COPD HBECs promoted differentiation into club, goblet and ciliated cells. Combined, these data suggest the impaired capacity of COPD cells to generate a normal airway epithelium is a reversible phenotype that can be regulated by the NOTCH3 target HEYL.

In contrast to the above findings that suggest decreased NOTCH3 signalling may contribute to the development of airway epithelial remodelling associated with CS exposure and COPD, our recent study demonstrated that in vitro CS exposure activates NOTCH3 signalling to promote development of GCMH in both non-smoker and COPD airway epithelial cells (Ref. 59). Cigarette smoke extract (CSE) exposure of in vitro ALI cultures of differentiated human mucociliary airway epithelium generated from primary non-smoker and COPD smoker human HBECs resulted in a decrease in the number of SCGB1A1+ club cells with a parallel increase in MUC5AC+ goblet cells, characteristic of GCMH (Ref. 59). Development of CSE-dependent GCMH corresponded with increased activation of the NOTCH3 receptor (i.e., increased NICD3 levels and nuclear localisation) with no change in the expression of NOTCH3 mRNA, suggesting that CSE regulates NOTCH3 protein levels post-transcriptionally. Importantly, inhibition of NOTCH3 signalling via treatment with the *γ*-secretase inhibitor dibenzazepine (DBZ) or siRNA-mediated knockdown of *NOTCH3* expression suppressed CSE-induced GCMH phenotype. In support of our findings, CS exposure increased the activation of NOTCH3 protein in human lung adenocarcinoma both in vitro and in vivo (Ref. 80). Furthermore, Gomi *et al*. (Ref. 81) demonstrated that long-term (28 days) over-expression of NICD3 in normal HBECs on in vitro ALI culture induced a phenotype characteristic of GCMH. Therefore, targeting NOTCH3 activity could be a novel therapeutic strategy to control GCMH in smokers with and without COPD. However, based on the knowledge that airway epithelium of smokers and COPD patients contain reduced levels of NOTCH3 mRNA and protein relative to healthy controls (Ref. 52), future work is required to better understand the cell type-specific expression pattern of NOTCH3 and the mechanisms regulating its activation state and kinetics in the context of COPD-associated airway epithelial remodelling.

Although, there is no report showing the direct role of NOTCH3 signalling in COPD-associated emphysema, a recent study described that enhanced NOTCH3 signalling contributes to Marfan syndrome-associated pulmonary emphysema in mice (Ref. 82). Marfan syndrome is a genetic disorder caused by mutations in the fibrillin-1 gene (Refs 82, 83, 84). Apart from other systemic effects, one of the major disease manifestations is altered lung function and pulmonary emphysema (Refs 82, 85, 86). The mouse model of Marfan syndrome (mgR mice) shows a progressive development of airspace enlargement (emphysematous changes), which correlates with an increase in NOTCH3 activation (but not NOTCH1, 2 or 4). Moreover, treatment with DAPT, a *γ*-secretase inhibitor which blocks global NOTCH signalling, prevented emphysema development in mgR mice, while decreasing NOTCH3 activation, thereby suggesting that NOTCH3 activation drives emphysema development in mgR mice (Ref. 82). However, the use of a global NOTCH signalling inhibitor questions the specificity of the NOTCH3-dependent effects. Therefore, future studies using strategies to specifically block NOTCH3 signalling in the mgR mouse model are required to strengthen and confirm the pathogenic role of NOTCH3 activation in driving emphysema development in Marfan syndrome.

### Viral exacerbations and COPD

Acute exacerbations caused by viral infections can result in significant morbidity, mortality and hospitalisations in COPD subjects (Refs 37, 38, 39, 87). RV is a common respiratory pathogen associated with increased GCMH in COPD subjects, resulting in severe and prolonged respiratory distress and airflow obstruction (Refs 40, 41, 42, 60). These symptoms are attributed to an increase in both virus-induced mucin production, and an increase in number of mucus-producing secretory (goblet) cells (Refs 40, 41, 42, 60, 87). A recent study by Jing *et al*. (Ref. 60) showed that in vitro RV infection of COPD cells differentiated on ALI culture resulted in the activation of NOTCH3 and its downstream target, HEY1. This NOTCH3-HEY1 activation correlated with increased mucin gene expression, with a parallel increase in the numbers of goblet cells (i.e., GCMH). Furthermore, they demonstrate that inhibition of NOTCH3 signalling via treatment with the *γ*-secretase inhibitor DAPT or shRNA-mediated knockdown of NOTCH3 expression suppressed RV-induced GCMH in COPD cells. Interestingly, RV infection of ALI differentiated epithelium from normal cells did not lead to increased NOTCH3 signalling, and development of GCMH, suggesting that COPD cells have inherent or intrinsic changes that make them susceptible to RV-dependent induction of NOTCH3 signalling. In contrast to our findings with CSE (Ref. 59), Jing *et al*. do not observe a change in the number of club cells upon RV infection, suggesting that the increase in the number of goblet cells may result from direct differentiation of BC into goblet cells, whereas CSE exposure leads to differentiation of club to goblet cells. Therefore, future studies, which could include lineage tracing experiments, are required to better understand the context-dependent mechanisms driving NOTCH3 activation in response to environmental stimuli (i.e., CS and viral) and the specific cell types involved in the development of GCMH.

### Lung cancer

Lung cancer is among the predominant causes of death worldwide and takes the top spot in cancer-related deaths (Refs 88, 89). Both COPD and lung cancer are CS-related diseases and are described as risk factors of each other, while commonly occurring as co-morbid conditions (Refs 90, 91, 92). Since NOTCH signalling is one of the key regulators of cell fate, with intricate control over cell proliferation, survival, differentiation and apoptosis, it is unsurprising that NOTCH is strongly related to lung cancer (Refs 54, 93, 94, 95, 96). Although NOTCH signalling is implicated in both small cell lung cancer (SCLC) and non-small cell lung carcinoma (NSCLC), NOTCH3 is primarily over-expressed in NSCLC (Ref. 97), which constitutes almost 85% of all lung cancer cases (Ref. 93). Furthermore, CS exposure increased the activation of NOTCH3 protein in human lung adenocarcinoma (a subtype of NSCLC) both in vitro and in vivo (Ref. 80). Interestingly, NOTCH3 plays a tumour-promoting role in NSCLC, while it acts as a tumour-suppressor in SCLC, suggesting cell type-specific functional roles of NOTCH3 in lung cancer (Ref. 93). A 5-year study to evaluate NOTCH3 expression in NSCLC patients undergoing surgical treatment showed that NOTCH3 was highly expressed in 51% of the NSCLC patients (Ref. 98). Moreover, survival of patients with higher expression of NOTCH3 was shorter as compared to patients with normal NOTCH3 levels, suggesting a direct correlation of NOTCH3 expression with lung cancer-related mortality (Ref. 98). Mechanistically, NOTCH3 signalling has been implicated in lung cancer metastasis (Refs 93, 96, 99). Activation of WNT signalling via Wnt3a ligand treatment upregulated the mRNA and protein expression of NOTCH3, and its downstream targets HEYL and HES1, while promoting cell invasion and anchorage-independent growth (Ref. 100). Additionally, knockdown of NOTCH3 abrogated the effects of Wnt3a treatment on cell invasion and epithelial mesenchymal transition (EMT)-like morphological changes, suggesting NOTCH3 is required for Wnt3a-mediated metastatic effects in NSCLC cells (Ref. 100). Overall, these studies provide strong clinical and mechanistic evidence of the pathogenic role of NOTCH3 signalling in NSCLC, and strengthen the rationale for therapeutically targeting its activity to treat the disease.

### Asthma

Asthma is an allergen-induced chronic lung condition marked by chronic airway inflammation, mucus hypersecretion, airway remodelling and obstruction, and increased airway hyper-reactivity (Refs 101, 102). A recent study by Reid *et al*. (Ref. 61) identified a potential role of NOTCH3 hyperactivation in mucus production associated with asthma. The authors found that NOTCH3 protein levels and nuclear staining (i.e., indicative of NOTCH3 activation) were increased in human bronchial sections from asthma subjects as compared to non-asthma. In support of this, NOTCH3 levels (mRNA and NICD3 protein levels) were similarly elevated in the in vitro ALI-differentiated airway epithelium generated from HBECs of asthmatics as compared to HBECs from non-asthmatics. In addition, increased NOTCH3 levels in asthmatic epithelium appeared more intense around areas of MUC5AC+ goblet cells. The authors further demonstrate that inhibition of NOTCH signalling using DBZ reduced in vitro MUC5AC expression and secretion in ALI cultures and subsequent siRNA-mediated knockdown of NOTCH3 expression showed a decrease in MUC5AC production. Combined, these data suggest that NOTCH3 activation in the airway epithelium drives MUC5AC expression and secretion and thus contributes to the increased mucus production in asthmatic airways. However, in contrast to the above study, a recent paper by Carrer *et al*. (Ref. 103) showed that blocking NOTCH2 (but not NOTCH3) activation using antisense oligonucleotides (ASOs) reduced house dust mite (HDM) induced GCMH in adult mouse lungs. The differences observed between these studies may reflect differences in the underlying mechanisms driving asthma-associated GCMH versus HDM-associated GCMH, and species-specific differences between the human and mouse airway epithelium, including the cell type-specific expression of NOTCH receptors and ligands (Refs 3, 45).

In addition to its role in regulating the airway epithelium, NOTCH signalling also regulates the balance of T-helper (Th) 1 and Th2 immune cells, which plays a crucial role in the pathogenesis of allergic asthma (Refs 48, 55, 104, 105). Global suppression of NOTCH signalling using *γ*-secretase inhibitors reduces airway inflammation in the ovalbumin (OVA)-induced murine model, suggesting that NOTCH signalling may play a pro-pathogenic role in asthma (Ref. 104). A later study identified that the imbalance of Th17/Treg (regulatory) cells in children with allergic asthma correlated with an increase in NOTCH1 activity (Ref. 106). Furthermore, in a murine model of OVA-induced asthma, NOTCH signalling inhibition using *γ*-secretase inhibitors suppressed Th17 cell responses along with decreasing asthma features, suggesting the direct role of NOTCH signalling in regulating Th17 cell differentiation (Ref. 107). However, the underlying complexity in the role of NOTCH signalling in asthma is evident by the findings that constitutive activation of NOTCH3 signalling promotes the generation and expansion of asthma-protective Treg cells (Refs 48, 108). Additionally, over-activation of NICD3 in activated CD4 + T cells promoted Th1 differentiation, which is known to elicit a protective T cell response in asthma (Ref. 109). Overall, these studies highlight the complexity of NOTCH signalling in asthma pathogenesis, where it is evident that different NOTCH receptors (including NOTCH3) regulate pathogenic or asthma-protective responses in a context-dependent manner.

### IPF

IPF is a progressive, irreversible chronic lung disease with a high fatality rate (Refs 110, 111, 112). The disease is caused in response to repeated injury which leads to damage and subsequent destruction of the alveolar compartment which reduces the gas exchange capabilities of the lung (Refs 111, 112). Pathological features of IPF include impaired alveolar re-epithelisation, elevated extracellular matrix (ECM) deposition, increased myofibroblast proliferation, parenchymal remodelling and the appearance of honeycomb cysts (composed of airway epithelial BC and mucin producing secretory cells) in the distal airways, which ultimately combine to cause life-threatening destruction of lung architecture (Refs 62, 113, 114, 115).

In general, activation of NOTCH signalling via different receptors is associated with factors that promote pulmonary fibrosis, such as myofibroblast differentiation, EMT, activation of TGF*β* and Wnt/*β*-catenin signalling, and increased proliferation and de-differentiation of alveolar epithelial type II cells (Refs 62, 116, 117, 118, 119, 120). However, a recent study by Carraro *et al*. (Ref. 63) characterizing the transcriptome of single cells from normal human lung versus lung tissue of patients with end-stage IPF identified alterations in the subsets of airway BCs, with expansion of a secretory primed population of BCs in the IPF lung that is capable of differentiating into mature mucus-producing goblet cells. The authors further demonstrate that NOTCH3 signalling activity is required to maintain this population of secretory primed BCs, and inhibition of NOTCH3 signalling with a NOTCH3-specific blocking antibody promoted their differentiation into goblet cells. These findings contrast with the previous studies from our lab demonstrating that inhibition of NOTCH3 signalling prevented CSE-mediated induction of goblet cell differentiation (Ref. 59). Potential reasons for the discrepancy between these studies maybe related to differences in the in vitro ALI culture systems and time points analysed, the method of inhibiting NOTCH3 activity (i.e., blocking antibody versus siRNA) and HBEC populations (i.e., cell sorted for specific populations versus no cell sorting) used for ALI culture which may respond differentially to either NOTCH3 inhibition or CSE treatment. Therefore, further studies are required to clarify the role of NOTCH3-dependent differentiation of individual airway epithelial cell populations and the timing of NOTCH3 signalling events that regulate these processes. In addition to its role in regulating airway epithelial cell differentiation in the context of IPF, a recent report by Vera *et al*. (Ref. 62) showed the specific role of NOTCH3 in fibroblast activation and development of pulmonary fibrosis. The authors demonstrate that bleomycin treated *Notch3^−/−^* mice have much smaller numbers of myofibroblasts and were protected from development of pulmonary fibrosis. More importantly, *Notch3^−/−^* mice showed less collagen deposition and improved lung function post bleomycin treatment, suggesting that targeting NOTCH3 might be a useful strategy to mitigate the lung function decline in IPF. Although this study provides significant evidence of the role of NOTCH3 in IPF development, it does not delve into the possible upstream causes of NOTCH3 activation. However, a previous study by Lai *et al*. (Ref. 121) demonstrated that reactive oxygen species-dependent activation of p38, JNK1/2 and NOTCH3 promoted basal and TGF-*β*1 induced differentiation and expression of ECM proteins in primary human lung fibroblasts (IMR-90 cells) in vitro. Moreover, TGF-*β*1 induced the expression of *α*-smooth muscle actin (a marker of myofibroblasts) and NOTCH3, both of which were suppressed by treatment with DAPT or NOTCH3-specific siRNA. Overall, these studies provide a strong rationale for targeting NOTCH3 signalling as a potential therapeutic strategy in controlling IPF.

### PAH

PAH is a rare, progressive and devastating disease in which there is high blood pressure due to thickening and narrowing of the small arteries in the lungs (Refs 122, 123). The blockage in the pulmonary vessels may progress to right-side heart failure which is the primary cause of high morbidity and mortality associated with PAH (Refs 124, 125, 126). At the cellular level, the main changes in PAH include proliferation of fibroblasts, infiltration of immune cells and proliferation of PASMCs (Refs 125, 127, 128, 129, 130). The above factors mediate the development of a vascular remodelling phenotype, called ‘neointimal lesions’, resulting in the elevation of pulmonary vascular resistance and ultimately heart failure (Refs 64, 131, 132, 133). There have been several studies investigating the role of NOTCH3 signalling in PAH, which are reviewed in detail by Morris *et al*. (Ref. 56). A report by Li *et al*. (Ref. 65) showed that PAH is characterised by elevated expression of NOTCH3 in PASMCs, and the severity of disease in both humans and rodents correlates with the amount of NOTCH3 present in the lungs. The development of neointimal lesions observed in PAH is thought to originate from the massive clonal expansion of a small number of smooth muscle cells, termed as the neointimal founder cells (Ref. 64). A recent and interesting study found that a minor subpopulation of NOTCH3 + VSMCs acts as the neointimal cell of origin in multiple mouse models of PAH (Ref. 64). Furthermore, studies demonstrate that overexpression of NOTCH ligand JAGGED-1 (JAG1) in human small PASMCs promotes their proliferation through activation of NOTCH3 signalling (Ref. 66). Inhibition of NOTCH signalling using DBZ abrogates the selection of this NOTCH3-marked neointimal founder cell subpopulation, resulting in significant improvement in pulmonary artery pressure in mouse models of PAH (Ref. 64). Therefore, identification of a distinct subpopulation of NOTCH3+ cells among normal tissues, which specifically generates neointimal lesions, provides novel avenues for therapeutic development in PAH.

Exposure to chronic hypoxia results in development of the vascular remodelling phenotype implicated in PAH (Refs 134, 135). In sync with the disease promoting role of NOTCH3 in PAH, *Notch3^+/−^* and *Notch3^−/−^* mice were resistant to PAH development compared to wild-type mice after 6 weeks of chronic hypoxia (Ref. 65). Furthermore, over-expression of the activated intracellular domain of NOTCH3 (NICD3) led to proliferation of PASMCs, which correlated with increased expression of the NOTCH downstream target Hes1, and decreased p27Kip1 expression (Ref. 65). The authors also confirmed that HES1 lies downstream of NOTCH3 signalling, as siRNA-mediated knockdown of HES1, prevented the proliferative effects of NICD3 over-expression. A more recent study (Ref. 67) provides evidence that elevated levels of NOTCH3 in PAH are regulated by sphingosine-1-phosphate-dependent signalling via the sphingosine-1-phosphate receptor 2. Moreover, a genetic basis of NOTCH3 activation in PAH was demonstrated by a recent study which showed that only the male mice with CADASIL (cerebral autosomal dominant arteriopathy with subcortical infarcts and leukoencephalopathy) causing mutation R169C (TgNotch3R169C), demonstrate gain-of-function NOTCH3 activation and develop PAH-like features (Ref. 68). Thus, the NOTCH3 R169C mutation may be associated with PAH susceptibility in males.

The above studies provide substantial evidence of the pathogenic role of NOTCH3 activation in PAH, thus making it a potential therapeutic target to control PAH. In support of this, a recent study by Zhang *et al*. (Ref. 66) using both mouse and rat models of PAH demonstrated that treatment with anti-NOTCH3 antibody (Ab 28042) which inhibits JAG1-dependent activation of NOTCH3 signalling, reversed PAH. Furthermore, treatment of animals with the anti-NOTCH3 antibody did not lead to local or systemic toxicity, suggesting that blocking JAG1-dependent activation of NOTCH3 signalling could be a promising therapeutic strategy for treating patients with PAH.

## Therapeutic strategies for targeting NOTCH3 signalling

Evidence provided in this review highlights the involvement of NOTCH3 signalling in the development of acute and chronic lung disease. Therefore, strategies to specifically target NOTCH3 signalling provide an attractive therapeutic option to treat and control lung disease pathogenesis (Refs 59, 60, 61, 62, 63, 64, 65, 68, 136). Several clinical studies have used *γ*-secretase inhibitors to block NOTCH signalling in various human diseases (Refs 137, 138, 139, 140), but the strategy lacks specificity for individual receptors, which can lead to global suppression of signalling and toxic side effects (Refs 141, 142, 143, 144). Therefore, more specific strategies to target NOTCH3 are required. Antibody-based inhibition is a clinically proven therapeutic strategy to selectively inhibit specific NOTCH receptor signalling (Refs 144, 145). To this end, blocking antibodies which suppress NOTCH3 activation via targeting the NOTCH3 NRR and HD (Ref. 146), or NOTCH3 ligand interactions (Ref. 66) have been successfully developed. These antibodies have been shown to provide anti-cancer activity and reverse pathology associated with IPF and PAH using in vitro and in vivo pre-clinical models of disease (Refs 66, 144). While blocking NOTCH ligands maybe less specific than directly targeting the receptor, this approach has proved successful in a pre-clinical murine asthma model, whereby blocking antibodies targeting the JAG1 and JAG2 ligands reversed OVA-induced GCMH (Ref. 147).

In addition to blocking antibodies, targeting of NOTCH3 expression with ASOs may provide an alternative strategy to specifically inhibit NOTCH3 signalling (Refs 103, 148, 149). Down-regulation of *Notch3* expression via systemic administration of *Notch3*-specific ASOs in a Notch3 gain-of function mutation (*Notch3^em1Ecan^*) mouse model of lateral meningocele syndrome ameliorates cortical osteopenia associated with the mice (Ref. 149). Therefore, while targeting of *Notch3* expression with ASOs failed to reduce HDM-induced GCMH in adult mouse lungs (Ref. 103), this therapeutic approach may be more applicable for lung diseases like IPF and PAH where the role of NOTCH3 signalling in disease pathogenesis is more clearly defined.

## Conclusions

In summary, there is rapidly accumulating evidence that alteration of NOTCH3 signalling in the adult human lung plays an important role in the development and pathogenesis of multiple acute and chronic lung diseases, including COPD, viral infections, lung cancer, asthma, IPF and PAH. Furthermore, both in vitro and in vivo pre-clinical models of lung disease have been utilised to successfully demonstrate the potential of targeting NOTCH3 signalling activity as a viable therapeutic strategy for treatment of human lung disease. However, it is important to study, modulate and target NOTCH3 signalling in a cell-specific manner in order to prevent off-target effects. Thus, further investigations are required to better understand at the disease and cell-specific level, the precise mechanisms whereby dysregulation NOTCH3 signalling leads to pathogenic outcomes. Success in these studies should lead to further improvement of existing therapeutic strategies to target NOTCH3 signalling and ultimately facilitate the development of new treatments for human lung disease.
